# Minimization of Intrinsic Impurity Concentration in ZnGeP_2_ Single Crystals via Directional Recrystallization

**DOI:** 10.3390/ijms27114890

**Published:** 2026-05-28

**Authors:** Alexander Gribenyukov, Alexey Lysenko, Nikolay Yudin, Elena Slyunko, Sergey Podzyvalov, Mikhail Zinovev, Vladimir Kuznetsov, Andrey Kalsin, Andrei Khudoley, Houssain Baalbaki, Maxim Kulesh, Alexey Olshukov

**Affiliations:** 1 Scientific Educational Center “Optical and Photonic Technologies”, National Research Tomsk State University, 634050 Tomsk, Russia; alexander.gribenyukov@yandex.ru (A.G.); festality@yandex.ru (A.L.); rach3@yandex.ru (N.Y.); cginen@yandex.ru (S.P.); muxa9229@gmail.com (M.Z.); robert_smith_93@mail.ru (V.K.); andrejkalsin@gmail.com (A.K.); houssainsyr1@gmail.com (H.B.); makksim13@gmail.com (M.K.); olshukov@mail.ru (A.O.); 2 Institute of Atmospheric Optics, Siberian Branch, Russian Academy of Sciences, pl. Academician Zueva, 1, 634055 Tomsk, Russia; 3 A.V. Luikov Heat and Mass Transfer Institute, National Academy of Sciences of Belarus, 15 P. Brovka Str., 220072 Minsk, Belarus; khudoley@hmti.ac.by

**Keywords:** ZnGeP_2_, chalcopyrite semiconductor, directional recrystallization, pre-growth purification, mid-infrared nonlinear optics, impurity phosphides, laser-induced damage threshold

## Abstract

Zinc germanium phosphide (ZnGeP_2_) is an important nonlinear crystal for mid-infrared conversion, but its performance is limited by residual absorption and intrinsic impurity phases. In this study, polycrystalline ZnGeP_2_ was synthesized by a modified two-temperature method, purified by inclined directional recrystallization for up to three cycles, and then grown into single crystals by the vertical Bridgman method. The resulting material was examined by shadow-projection imaging, transmission spectroscopy in the 650–2500 nm range, absorption measurements at 2.097 µm, laser-induced damage threshold (LIDT) testing, and powder X-ray diffraction. Repeated purification improved optical homogeneity and near-infrared transparency, while the absorption coefficient at 2.097 µm decreased from 0.45 to 0.30 cm^−1^ after three purification cycles. Semi-quantitative PXRD analysis showed progressive suppression of intrinsic impurity phosphides, with phase purity increasing from 86.31% after the first cycle to 95.995% after the second and reaching 100% after the third within the detection limit of the method. However, the LIDT decreased with increasing purification number, indicating a trade-off between lower optical losses and damage resistance. These results demonstrate that inclined directional recrystallization is an effective pre-growth purification route for ZnGeP_2_ and that the optimal number of purification cycles should be selected according to the intended application.

## 1. Introduction

Zinc Germanium diphosphide (ZnGeP_2_, hereinafter referred to as ZGP) belongs to the family of chalcopyrite semiconductors and is one of the fundamental crystals for nonlinear optics in the infrared (IR) range, owing to its combination of high nonlinear efficiency and the possibility of phase matching enabled by birefringence across a broad spectral range from 3 to 12 µm [1,2,3]. ZGP is a key material for parametric frequency converters in the mid-IR range when pumped by laser radiation near a wavelength of 2 µm [1,2]. The practical demand for ZGP is largely driven by tasks involving parametric frequency conversion in the wavelength range of approximately 3–8 µm under pumping by well-developed and reliable laser sources emitting around 2 µm [4,5]. At the same time, the requirements for ZGP crystal quality in the context of nonlinear frequency conversion are particularly stringent: even moderate residual absorption and local defects lead to thermal effects, degradation of the spatial characteristics of the beam, and a reduction in the laser-induced damage threshold (LIDT). Consequently, issues related to pre-growth purification of the polycrystalline batch and optimization of growth conditions for synthesized polycrystalline ZGP are decisive for the optical and spectral characteristics of the material [5,6,7].

One of the main limitations for ZGP remains the presence of undesirable absorption in the pump wavelength region below 2 µm. This absorption is associated both with impurity centers caused by point defects (Zn and P vacancies, substitutional defects) and with intrinsic defects of the crystal lattice, as well as with stoichiometric deviations and ordering defects [6,7,8]. Studies devoted to the role of dopant impurities and defect states in limiting crystal transmission in the 1–2 µm wavelength range emphasize that absorption near the band edge and within the operational wavelength region can be induced by lattice defects, while non-stoichiometric melts and processes accompanying crystal growth serve as sources of donor-acceptor centers [6]. It is additionally noted that incomplete removal of structural disorder and features of order–disorder transitions in ZGP can lead to the formation of defects affecting optical properties [6,7].

For defectoscopy of ZGP crystals, methods such as X-ray topography and optical visualization of bulk inhomogeneities are actively employed, enabling the detection of dislocations, growth striae, inclusions, and microdefects [9,10,11]. Since growth inhomogeneities are accompanied by local variations in composition and refractive index, they can degrade the quality of the output beam generated within the crystal and induce effects critical for nonlinear conversion (e.g., astigmatism and beam geometry distortion) [5].

Previous studies have shown that growth striae in ZGP single crystals are associated with the accumulation of intrinsic binary phosphides at the crystallization front and are accompanied by near-surface pores; furthermore, compositional differences at the level of local inclusions are observed between light and dark bands [5]. Importantly, such inhomogeneities can significantly distort the laser beam profile and alter the magnitude of nonlinear refraction, whereas their impact on laser damage resistance may be less pronounced (within comparisons of growth regimes) [5]. On the other hand, the LIDT of ZGP demonstrates sensitivity to the structural quality of the lattice and to laser exposure parameters (pulse duration, repetition rate), indicating the necessity of a comprehensive approach: reducing optical losses should be accompanied by decreased defect density and stabilization of growth conditions [12].

A fundamental technological challenge in working with ZGP is the combination of the multicomponent nature of the Zn–Ge–P system and the high volatility of zinc and phosphorus, which complicates the maintenance of stoichiometry and promotes the formation of secondary phases [5]. It is noted that, in the presence of two highly volatile components, strictly stoichiometric growth is practically unattainable; moreover, ZGP is further characterized by a large number of binary compounds within this system, making the production of homogeneous crystals with specified optical properties a nontrivial task [5]. In this regard, approaches to synthesizing the initial polycrystalline material and controlling partial pressures within the synthesis ampoule are of great importance. The two-temperature synthesis method for ZGP allows control over the formation conditions of the ternary compound, reduces risks associated with high phosphorus pressure, and yields material suitable for subsequent crystal growth [13]. Earlier studies also addressed issues related to the synthesis and melt growth of ZGP and associated limitations, including the influence of the gas phase and growth regimes determining phase composition and defectivity [14]. ZGP growth is implemented via various melt crystallization techniques, including the classic vertical Bridgman method and directional crystallization schemes in a temperature gradient. To enhance optical quality, the literature describes approaches employing an “ultra-low” temperature gradient during horizontal growth (HGF), aimed at reducing thermomechanical stresses, decreasing defect density, and improving optical characteristics [4]. Classical works on the growth of IIB–IVA–VA_2_-type compounds (including ZnGeP_2_) also highlight the necessity of fine-tuned control over crystallization conditions when growing these “diamond-like” compounds [15].

High purity of starting substances and/or the synthesized compound is a fundamental prerequisite for obtaining crystals with low optical losses, especially in the IR range, where absorption contributions from impurities and defects manifest most critically for nonlinear-optical applications [7,16]. Modern reviews on high-purity material technologies emphasize that advances in electronics, photonics, and vacuum technology stimulate the refinement of deep purification methods, including processes based on segregation during crystallization and multiple cycles of impurity redistribution [16].

Zone melting is one of the primary methods for deep material purification, relying on differences in impurity distribution coefficients between solid and liquid phases; process efficiency depends on the distribution coefficient, molten zone length, degree of melt mixing, and zone travel speed [17,18,19]. Educational literature on semiconductor materials notes that mixing within the molten zone can be hindered; therefore, the zone travel speed should be comparable to the impurity diffusion rate, otherwise inhomogeneous impurity distribution arises [17]. Modern modeling studies of zone melting demonstrate that thermal and kinetic parameters, as well as evaporation processes (for volatile impurities), significantly affect purification outcomes and the growth of homogeneous single crystals, necessitating process optimization [20]. Simultaneously, for multicomponent compounds containing highly volatile components, zone melting is complicated by the need for strict control over composition and partial pressures, as well as reduced process stability: any loss of a volatile component leads to non-stoichiometry, formation of secondary phases, and defects [5]. Therefore, when selecting a purification methodology for polycrystalline ZGP charge, priority is typically given to schemes ensuring hermetic sealing, a controlled temperature profile, and minimization of Zn and P losses.

Directional crystallization is widely applied both for crystal growth and purification: The work [17] emphasizes that single-crystal growth by this method is usually combined with material purification. In sealed directional recrystallization or gradient-freeze purification, impurity redistribution is governed by segregation at the moving solid–liquid interface and by the combined influence of diffusion, natural convection, inclination-induced flow, and forced mixing due to ampoule rotation. In the present inclined rotating configuration, the inclination and axial rotation are used to reduce stagnant regions and stabilize impurity rejection toward the tail part of the ingot. For zone melting schemes with a temperature gradient, technological advantages are also noted: absence of mechanical zone movement, the possibility of crucible-free processing, and reduced likelihood of spontaneous nucleation due to small melt volume and lack of stirring [18].

Separately, the Bagdasarov method deserves mention as a variant of directional crystallization in a boat configuration, involving growth from a seed, visual monitoring, and formation of a temperature field ensuring directional growth [21]. It is noted that a small melt height combined with a large free surface area can reduce convective flow intensity and enhance the removal efficiency of certain impurities (including via evaporation), while repeated recrystallization cycles can provide additional chemical purification of the material [21]. These features are methodologically important for pre-growth purification tasks, where the objective is not only to form a large ingot but also to deliberately reduce impurity concentrations and the likelihood of secondary phase formation.

Considering the literature data presented above, the selection of a pre-growth purification technology for polycrystalline ZGP charge must simultaneously satisfy several criteria: (1) minimization of Zn and P losses (and, consequently, suppression of non-stoichiometry); (2) effective segregation of impurities and secondary phases; (3) suppression of conditions promoting the formation of growth striae and local compositional inhomogeneities that affect beam quality and nonlinear-optical characteristics [5,7,12]. The work [5] on ZGP synthesis and growth demonstrate that growth striae and banded compositional inhomogeneity are associated, among other factors, with the accumulation of binary phosphides at the crystallization front and compositional variations during growth, which negatively impact spatial transmission characteristics and nonlinear interaction. Consequently, pre-growth purification aimed at reducing impurity content and “intrinsic” phosphide inclusions represents a direct technological tool for improving optical parameters in the near-IR region.

Zone melting, as a deep purification method, is potentially effective for many materials; however, for multicomponent systems with highly volatile components (such as ZGP), practical limitations arise related to maintaining composition and preventing volatile losses. Moreover, modeling of zone melting shows sensitivity to thermal and kinetic parameters, including evaporation processes [20]. 

The modification of inclined directional crystallization scheme proposed in this work for pre-growth purification of ZGP appears most rational based on the combination of factors: it retains the key advantages of directional crystallization (controlled growth front, reduced convection role at small melt height, possibility of multiple cycles), while being technologically oriented toward enhancing segregation efficiency and mechanical separation of the “tail” section of the ingot, where impurities and secondary phases concentrate. Given that growth striae and compositional inhomogeneity can significantly distort the laser beam profile and affect nonlinear refraction parameters [5], systematic investigation of the influence of the number of such purification cycles and subsequent growth parameters on absorption/transmission coefficients in the near-IR region and on LIDT represents a timely and methodologically justified continuation of existing research [5,7,12,13,14].

## 2. Results and Discussion

To visually compare transparency and the presence of bulk inhomogeneities in crystals grown after 1–3 purification cycles, longitudinal sections were examined. Visualization of the interior of the control sections was performed using a shadow-projection method in transmitted light under identical illumination conditions (a 20 W incandescent lamp). The images were recorded with a silicon CCD sensor.

**Figure 1:**
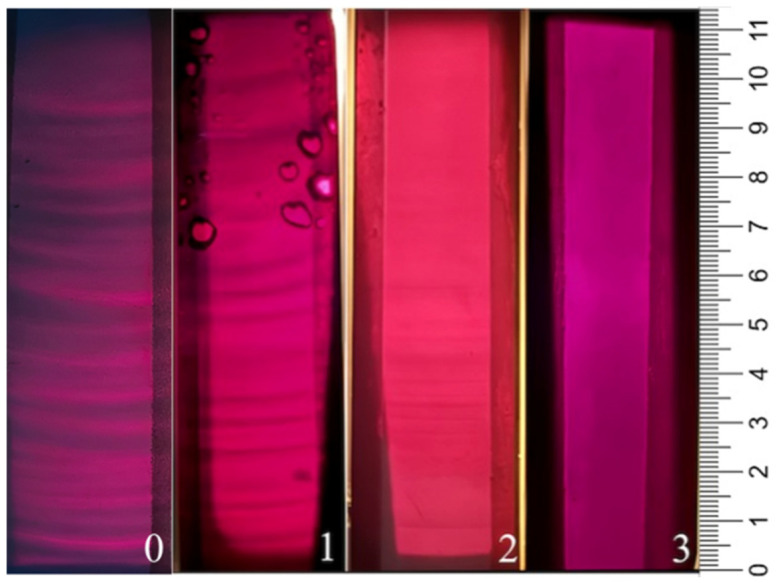
Shadow images of plate samples (cut along the growth axis) for different N of purification cycles.

Figure 1 shows the shadow images obtained in transmitted light for thin longitudinal cuts of single crystals taken along the crystal growth axis. The sample numbers correspond to the number of purification cycles the material underwent before crystal growth in the vertical Bridgman furnace. Number 0 corresponds to a sample that did not undergo inclined purification. Number 1 in Figure 1 corresponds to the shadow image of a single-crystal sample grown after one inclined-purification cycle. The shadow pattern shows a section that contains dark growth bands along its entire length, as well as clearly visible pores in the upper part of the crystal.

Growth bands form when a certain amount of impurity accumulates near the crystallization front of the growing single crystal. Once the impurity concentration reaches a threshold, it causes a local change in the crystallization temperature, which leads to the formation of a layer enriched with impurities. In zinc–germanium diphosphide, such impurities are variously valent zinc phosphides. The mechanisms of growth-band formation, as well as wall-adjacent pores, are described in detail in [5].

The obtained data allow us to conclude that the crystal purified three times demonstrates higher “transparency”/homogeneity in visual inspection compared with the crystal purified once; the material subjected to a single purification can be described as more defective. In the context of optical properties, such images serve as indicators of changes in the scattering contribution and/or local absorption associated with inhomogeneities (inclusions, banding, pores, secondary phases).

**Figure 2:**
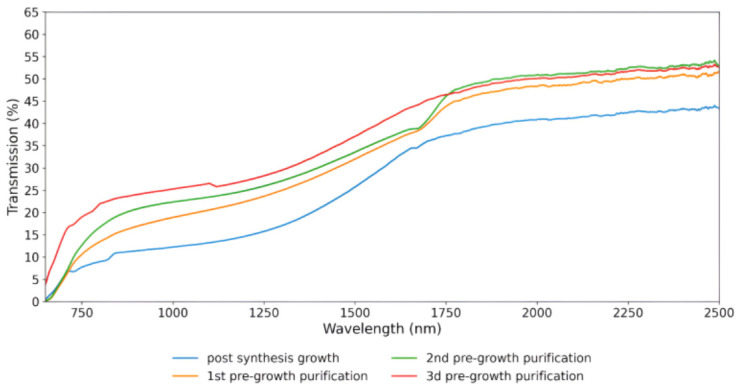
Transmission spectra of ZnGeP2 in the 650–2500 nm range for material immediately after synthesis and after successive pre-growth purification stages.

Figure 2 shows the transmission spectra of ZnGeP2 in the 650–2500 nm range for material grown immediately after synthesis and after successive pre-growth purification stages. All samples exhibit a rapid increase in transmission in the short-wavelength region, followed by a gradual rise toward the near-IR range, reaching a quasi-plateau at λ ≳ 2000 nm. This behavior is typical for materials whose primary losses are determined by the combined contribution of residual absorption and scattering.

The sample immediately after synthesis shows the lowest transmission across the entire spectral range, indicating elevated optical losses. Such losses may be caused by:

Residual impurities and/or secondary phases that introduce additional absorption channels;

Defect states and color centers that form sub-band absorption;

and Light scattering on micron-scale inhomogeneities (porosity, inclusions, microcracks), which is especially noticeable at shorter wavelengths.

Although the transmission spectra in the present work were measured in the 650–2500 nm range, ZnGeP_2_ is primarily used as an OPO medium for generating mid-infrared radiation, including the 3–8 µm range. Therefore, the presented optical characterization should be considered mainly as an assessment of transparency in the pump region and resistance to damage under pumping with Ho/Tm lasers around 2 µm. This region is technologically important, as the pump radiation typically has the highest intensity within the OPO crystal, and residual absorption at the pump wavelength can determine heat generation and potentially initiate laser-induced damage. For the generated radiation in the 3–8 µm range, propagation losses may also affect conversion efficiency; however, quantitative absorption in this range was not measured in the present work. Consequently, we do not extrapolate the measured dependencies from the 650–2500 nm range to the mid-IR region and consider direct FTIR measurements and OPO tests in the 3–8 µm range as a necessary direction for further research.

After the first pre-growth purification, a pronounced increase in transmission is observed throughout the range, consistent with a reduction in the concentration of absorbing centers and/or a decrease in the number of scattering inhomogeneities. The second purification leads to further enhancement of transparency and a higher “baseline” in the near-IR range, reflecting additional suppression of residual absorption (including possible intraband contributions from impurity and defect states). The maximum improvement is achieved after the third pre-growth purification: this sample exhibits the highest transmission and the most stable spectral behavior at λ ≳ 1800 nm, indicating the best optical homogeneity and minimization of optical losses.

Overall, these results confirm that sequential pre-growth purification effectively increases the transparency of ZnGeP_2_, likely due to the simultaneous reduction in sub-band absorption (impurity-defect related) and scattering on structural inhomogeneities.

The phase composition and structure of polycrystalline ZGP after purification were determined using X-ray diffraction (XRD). Samples were examined on a Rigaku MiniFlex 600 diffractometer (Rigaku Corporation, Tokyo, Japan) with a Cu X-ray tube (wavelength 1.541862 Å). The maximum power of the X-ray tube is 600 W. The diffractometer is equipped with a vertical goniometer with a scanning speed of 10°/min. The minimum goniometer step on the 2θ scale was 0.005°. Measurements were performed at 40 kV and 15 mA. Phase analysis was performed using the PDF-4+ database, containing over 444,100 entries of inorganic diffraction data, and the full-profile analysis software POWDER CELL 2.4. Scans were carried out over 3–60° with a step of 0.02°. X-ray structural analysis allowed investigation of the crystal structure by determining lattice parameters, symmetry, and atomic positions in the unit cell.

Semi-quantitative comparison of the phosphide-phase composition was performed by analyzing PXRD peak lists using integrated peak intensities (Int. I). All reflections attributed to Zn–P (Zn_x_P_γ_), Ge–P (Ge_x_P_γ_), and ZnGeP_2_ phosphide phases were included in the calculation. To avoid double-counting in multiphase attribution of a single peak, for each peak r with integrated intensity Ir corresponding to kr phases, the intensity Ir was equally divided among the phases, i.e., each phase received a share Ir/kr. The total intensity of each phase and the normalized fractions were then calculated, along with the impurity index Simp,I and the phase-purity index PurityI.

**Figure 3:**
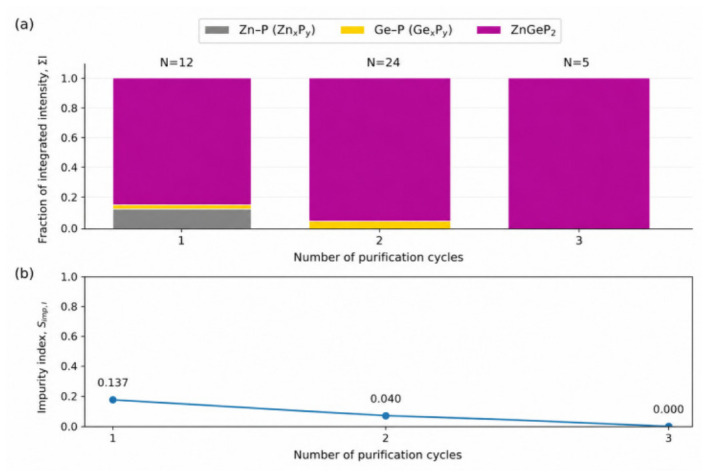
Semi-quantitative evolution of phosphide phases during purification derived from powder X-ray diffraction (PXRD) peak lists using integrated intensities. (**a**) Fractional contribution of phosphide phases calculated from the summed integrated peak intensities (ΣI). Only phosphide reflections were included. For peaks assigned to multiple phases, the integrated intensity was distributed equally among the assigned phases. N indicates the number of phosphide peaks included in the calculation for each purification cycle. (**b**) Integrated-intensity impurity index, S_imp,I = (ΣI_Zn–P + ΣI_Ge–P + ΣI_other)/ΣI_total, showing progressive removal of impurity phosphides with increasing purification cycles. Numbers 1, 2, and 3 on the x-axis denote the first, second, and third purification cycles, respectively.

Figure 3a illustrates the fractional contribution of the phosphide phases, calculated from the sum of integrated peak intensities (ΣI) identified in PXRD after 1–3 purification cycles. The columns are normalized to 1 (100%), thus representing the composition of the phosphide portion of the signal: Zn–P (Zn_x_P_γ_), Ge–P (Ge_x_P_γ_), and ZnGeP2.

If a single peak was attributed to multiple phases (multiphase attribution), its integrated intensity was divided equally among those phases (the 1/k rule). Above each column, N—indicates the number of phosphide peaks included in the calculation for that purification cycle. The figure shows the fraction of Zn_x_P_γ_ reflections.

Figure 3b shows the impurity index based on integrated intensities, Simp,I, which quantitatively describes the fraction of phosphide impurity contributions (Zn–P and Ge–P) in the total phosphide signal. A decrease in Simp,I with an increasing number of purification cycles indicates a sequential reduction in the relative contribution of impurity phosphides and a corresponding increase in the phase purity of the target ZnGeP2, according to this semi-quantitative assessment.

Total integrated intensity of phase i:

*I_i_* = *Σ_r_* (1*_i_*_∈_*_r_*/*k_r_*) *I_r_* (1)

where

*I_i_*—total integrated intensity of peaks assigned to phase *i*;
*r*—index of the peak in the PXRD list;
*I_r_*—integrated intensity of peak *r* (column Int. I);
*k_r_*—number of phases assigned to peak *r* (for single-phase peaks, *k_r_* = 1);
$1_{i\in r}$—indicator: 1 if peak *r* is attributed to phase *i*, 0 otherwise.

Total intensity of all phosphides:

*I_tot_* = *Σ_r_ I_r_* (2)

where *I_tot_* is the sum of integrated intensities of all peaks included in the analysis (phosphides only).

Normalized phase fraction by *ΣI*:

*S_i,I_* = *I_i_*/*I_tot_* (3)

where *S_i,I_* is the phase fraction contribution based on integrated intensity (within the phosphide portion).

Impurity index and phase purity index:

*S_imp,I_* = (*I_Zn–P_* + *I_Ge–P_* + *I_other_*)/*I_tot_* (4)

*Purity_I_* = 1 − *S_imp,I_* (5)

where

*S_imp,I_*—fraction of phosphide impurities (*Zn–P* and *Ge–P*) in the phosphide signal;
*Purity_I_* —phase-purity index of the target ZnGeP2 according to this metric.

According to the semi-quantitative analysis of the integrated peak intensities (ΣI), a monotonic decrease in the contribution of impurity phosphides is observed with an increasing number of purification cycles (Figure 3a,b). After the 1st purification cycle, the contribution of ZnGeP2 was 86.31%, with impurity contributions from *Zn–P* (12.04%) and *Ge–P* (1.65%); N = 12 phosphide peaks were included in the calculation. The impurity index was 0.1369, corresponding to a phase-purity index of 0.8631. After the 2nd purification cycle, no *Zn–P* contribution was detected in the peak list, while ZnGeP2 increased to 95.995% with a residual *Ge–P* contribution of 4.005%; N = 24. The impurity index decreased to 0.0400, and the phase purity increased to 0.9600. After the 3rd purification cycle, only the ZnGeP2 phase was observed among the phosphide peaks (100%); N = 5. The impurity index was 0.0000 and the phase-purity index was 1.0000, indicating the absence of detectable impurity phosphides within the sensitivity of the analysis.

o  After 1 purification: *S_imp,I_* = 0.1369 → *Purity_I_* = 0.8631 (86.31%).
o  After 2 purifications: *S_imp,I_* = 0.0400 → *Purity_I_* = 0.9600 (95.995%).
o  After 3 purifications: *S_imp,I_* = 0.0000 → *Purity_I_* = 1.0000 (100%).

The complete PXRD analysis tables can be found in Appendix A of this article.

**Figure 4:**
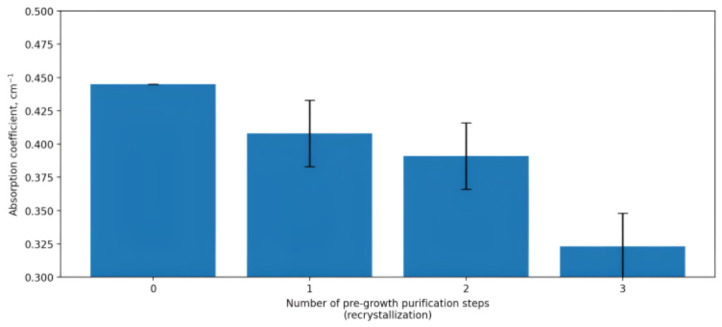
Dependence of absorption on the number of pre-growth purification cycles of ZGP.

Figure 4 presents the optical absorption values at a wavelength of 2.097 μm for samples that underwent different numbers of pre-growth purification cycles. The measurements were carried out using continuous-wave Ho:YAG laser radiation, taking into account multiple reflections from the faces of the samples under study.

To determine the LIDT of the dark and light regions of the crystals, the standard R-on-1 method was used. A Ho:YAG laser pumped by a continuous thulium fiber laser served as the source of test radiation with a wavelength of 2.097 μm. The Ho:YAG laser operated in the actively Q-switched regime with a pulse repetition rate of 20 kHz and a pulse duration of 35 ns. The amplitude of the nanosecond pulses, their duration and energy, as well as the beam diameter, were stable under constant pumping (random pulse-amplitude variations did not exceed 5%).

The LIDT measurements were performed with an exposure time of τex = 5. The sample under study was irradiated with packets of laser pulses at a fixed fluence level that did not cause surface damage to the crystals. The fluence was then increased in steps of ~0.1 J/cm^2^. When visible damage appeared on one of the surfaces of the nonlinear element, the experiment was stopped. The sample was then translated by 0.5 mm in height or width using a two-axis translation stage, and the experiment was repeated five times.

The probability of optical breakdown was determined by establishing the relationship between the cumulative probability and the optical-breakdown fluence. The optical breakdown threshold was taken as the fluence corresponding to the extrapolation of the breakdown probability to zero [22]. According to ISO 11254-2:2001 [22], the laser fluence was determined using the following expression:

(6) $$W=8\frac{P_{av}}{f\pi d^{2}}$$

where *d* is the diameter of the laser beam, *P_av_* is the average power of the laser radiation, and *f* is the pulse repetition rate.

**Figure 5:**
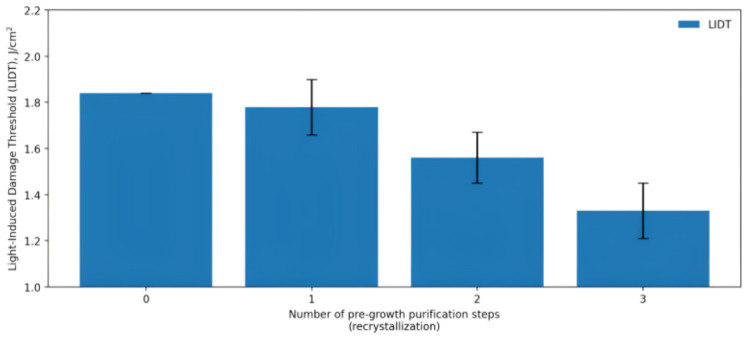
Dependence of LIDT on the number of pre-growth purification cycles, including the unpurified sample, N = 0.

Figure 5 shows a histogram of the dependence of the optical-damage threshold on the number of purification cycles to which the polycrystalline material was subjected prior to crystal growth. Here, the uncleaned sample was included in the LIDT comparison as N = 0. The resulting dependence indicates a monotonic decrease in LIDT over the investigated range of 0–3 cleaning cycles.

The results shown in Figure 5 demonstrate that an increase in the number of pre-growth purification cycles leads to a decrease in the laser-induced damage threshold (LIDT). This may be associated with the accumulation of technological contaminants introduced into the material during repeated high-temperature processing; it is difficult to completely eliminate such impurities during multiple purification cycles, which results in degradation of the laser damage resistance.

Examination of Figure 4 and Figure 5 shows that increasing the number of purification cycles leads to an ambiguous outcome: the average absorption coefficient decreases (favorable for optical applications), but LIDT decreases as well (critical for high-power laser operation).

However, the decrease in LIDT should not be directly attributed to residual *Zn–P* or *Ge–P* crystalline phases detected by PXRD. PXRD confirms the suppression of these phases only within the sensitivity limits of the method, whereas LIDT can be limited by rare localized damage centers, including trace contaminants, amorphous or nanoscale inclusions, surface and subsurface defects, local stress fields, as well as atomic-scale point defects. Since the elemental composition of these centers was not directly determined in the present work, further clarification requires highly sensitive analytical techniques such as ICP-MS, GDMS, SIMS, or XPS/EDS. Therefore, repeated high-temperature treatment may lead to the formation or accumulation of such localized centers even when the average absorption coefficient decreases and crystalline impurity phases are not detected by PXRD. As direct elemental analysis was not performed in the present study, the contribution of process-related contamination is considered a possible, but not exclusive or unequivocally proven, mechanism. Such a divergence of trends is consistent with the fact that the absorption coefficient and LIDT are governed by different types of defects: absorption reflects integral losses, whereas LIDT is often limited by relatively rare but particularly “dangerous” localized centers (micro-inclusions, technological contamination, local stresses, etc.) [5,6,7,8,9,10,11].

The observed dependence of the absorption at 2.097 µm and LIDT on the number of purification cycles indicates that repeated inclined directional crystallization enhances the redistribution and rejection of intrinsic impurities and secondary phases (which strongly affect absorption at wavelengths < 2.097 µm) into the tail of the ingot, followed by removal of this segment. At the same time, the inclined geometry may additionally facilitate spatial separation of inclusions/secondary phases (due to a combination of gravitational and hydrodynamic factors), thereby reducing the likelihood of their trapping by the crystallization front.

## 3. Materials and Methods

The study described in this article aims to determine the influence of the parameters and number of pre-growth purification cycles of ZGP performed by the inclined directional crystallization method on its optical properties in the near-IR region and on its laser-induced damage threshold (LIDT).

**Figure 6:**
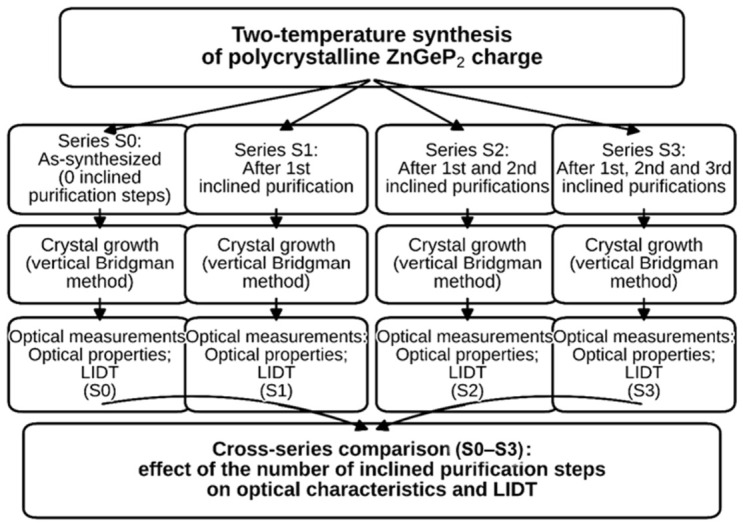
Block diagram of the experimental cycle (synthesis → N purifications → growth → sample preparation → measurements). Arrows indicate the sequence of processing and characterization steps.

The experimental procedure included the following sequence of steps:

Synthesis of polycrystalline ZnGeP_2_ using the two-temperature method;

Pre-growth purification of the polycrystal by inclined directional crystallization (0–3 purification cycles);

Growth of single crystals by the vertical Bridgman method using an oriented seed;

Fabrication of samples (plates/elements with specified orientation) and their polishing;

Investigation of the optical and structural characteristics of the samples (transmission/absorption in the near-IR range, LIDT, visualization of bulk defects, X-ray structural analysis), see Figure 6.

In the present work, ZnGeP_2_ was synthesized according to a published modified two-temperature method [13]. Zn and Ge were placed in the hot zone, while P was placed in the cold zone of the reactor. At the initial stage, the temperature of the hot zone was approximately 1000–1010 °C, and the temperature of the cold zone was about 500–520 °C; the dwell time under these conditions was 3 h. Such a hot-zone temperature, which is below or close to the melting point of ZnGeP_2_, was chosen intentionally: in the presence of free phosphorus, increasing the temperature above the melting point of ZnGeP_2_ can lead to a rise in pressure inside the ampoule and to the transport of volatile components, primarily zinc, into the gradient zone. After the phosphorus-binding reaction was completed, the temperature of the cold zone was raised to that of the hot zone over 2 h, which promoted the return of volatile binary phosphides from the gradient zone and prevented their condensation. The material was then held at 1050 °C for 6 h to homogenize the melt. Thus, the stage above the melting point pertained to melt homogenization rather than the initial stage of chemical synthesis. Unlike the original formulation, the term “modified two-temperature method” here refers to the published scheme in which the temperature of the cold zone is increased at the final stage; in the present work, only the charge mass was adapted. This approach enables the synthesis of comparatively large quantities of material (up to 500 g per process), whereas the single-temperature method is usually limited to significantly smaller batch masses (around 25 g) [5] and may involve hazardous situations due to the high vapor pressure of phosphorus at elevated temperatures.

ZGP synthesis proceeded through the intermediate formation of binary phosphides. In the 480–550 °C range, molten zinc reacted with phosphorus vapor to form zinc phosphide with a low phosphorus content [4]:

3 Zn(L) + 0.5 P_4_(G) → Zn_3_P_2_(S) (7)

where S, L, and G correspond to solid, liquid, and gas phases, respectively.

With increasing temperature and phosphorus pressure in the 550–850 °C range, the following reaction occurred:

Zn_3_P_2_(S) + P_4_(G) → 3 ZnP_2_(S) (8)

Because of the limited diffusion rate of phosphorus into solid zinc phosphide, the conversion of Zn_3_P_2_ to ZnP_2_ during heating was incomplete. At temperatures around 750 °C, the formation of germanium phosphide was observed:

Ge(S) + 1/4 P_4_(G) → GeP(L) (9)

Formation of the ternary compound begins at temperatures above 900 °C [8] and proceeded, in particular, via the reactions:

ZnP_2_(S) + Ge(L) → 3 ZnGeP_2_(S,L) (bulk reaction) (10)

Zn_3_P_2_(S) + 3GeP(L) + 1/4P_4_(G) → ZnGeP_2_(S,L) (surface reaction) (11)

After synthesis, the polycrystalline material was used as the feedstock for subsequent stages of pre-growth purification and single-crystal growth. After extraction, the material in the form of a compact polycrystalline ingot was cleaned of residual phosphorus using nitric acid, rinsed, and dried in a vacuum oven. This procedure minimized additional contamination between the synthesis and the subsequent stage of directional recrystallization.

**Figure 7:**
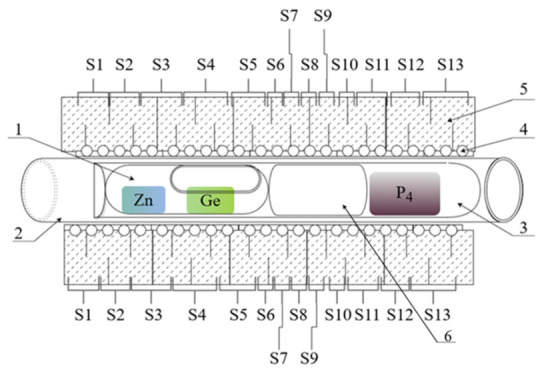
Schematic diagram of the thermal setup for two-temperature synthesis of ZGP: 1—crucible with reagents; 2—damping tube; 3—evacuated synthesis ampoule; 4—resistive heater; 5—lining; 6—quartz insert for zone separation; S1–S13—zones of individual temperature control.

Figure 7 shows the thermal setup used for the two-temperature synthesis of ZGP.

To minimize second-phase inclusions and deviations from stoichiometry in ZGP single crystals, a purification procedure for the ternary compound was applied prior to crystal growth. The method was based on an inclined thermal setup. The apparatus consisted of a tubular furnace mounted on a support frame at an angle to the horizontal. A metal tube made of heat-resistant steel was placed in the working zone of the furnace; the lower end of the tube was fixed in a rotation device. An inclination angle of approximately 12° is typically chosen as the optimal regime for thermogravitational mass transport in an ampoule during the synthesis and purification of ZnGeP_2_. At this tilt, a stable longitudinal gravitational component appears, facilitating drainage and redistribution of condensate/melt along the axis, reducing the probability of stagnant zones and local overheating, and thereby stabilizing the position of evaporation–condensation zones at a given temperature gradient. At the same time, the tilt remains small enough to avoid undesired convection and blurring of zone boundaries, which is crucial for reproducibility in composition and purity. Additionally, the inclination increases the effective area of the melt surface (to first order, the free-surface cross-section in a cylindrical ampoule increases roughly as 1/cos θ compared with the horizontal orientation), which can enhance the intensity of evaporation and gas-phase exchange. An angle of ~12° often proves to be a practical compromise—large enough to noticeably increase surface area, yet small enough to avoid issues such as wall exposure, melt streaks, or unstable film flow at realistic fill levels. Axial rotation disrupted the diffusion boundary layer ahead of the crystallization front and reduced the likelihood of impurity trapping, thereby increasing the efficiency of impurity rejection [23].

**Figure 8:**
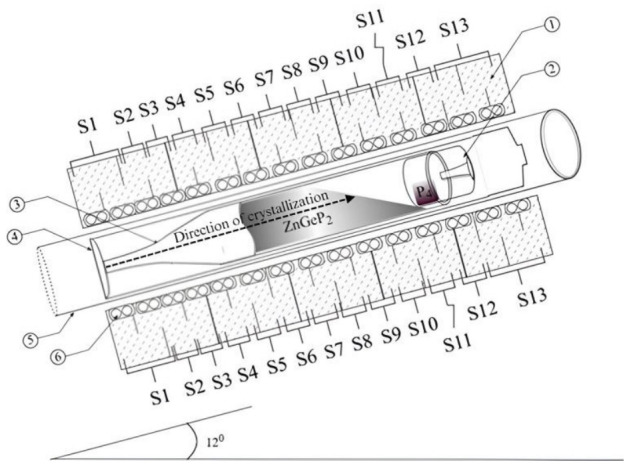
Diagram of the inclined directional crystallization setup: 1—lining; 2—phosphorus container; 3—crucible with conical bottom; 4—evacuated ampoule; 5—damping tube made of thermally conductive ceramics; 6—resistive heaters; S1–S13—zones of electronic temperature control.

The inclined-crystallization furnace consisted of several thermal, resistive sections enabling the required temperature gradient to be set. Axial variations of the gradient were electronically controlled by independently programming the target temperature of each thermal section. A conical crucible with an oriented seed and a batch of polycrystalline batch was placed inside a quartz-glass ampoule; the ampoule was then evacuated and loaded into the inclined thermal setup, schematically shown in Figure 8, to undergo the subsequent purification cycle.

The purification process included: (a) complete melting of the polycrystalline batch, and (b) slow longitudinal-axial crystallization onto the seed while the temperature gradient was translated along the furnace. During inclined recrystallization, solidification starts from the seed end of the crucible and proceeds toward the opposite end of the ingot. As a result, impurities were displaced toward the tail section of the ingot due to differences in the crystallization temperatures of the main phase and the inclusions. The main technological parameters of the purification processes are given in Table 1.

**Figure 9:**
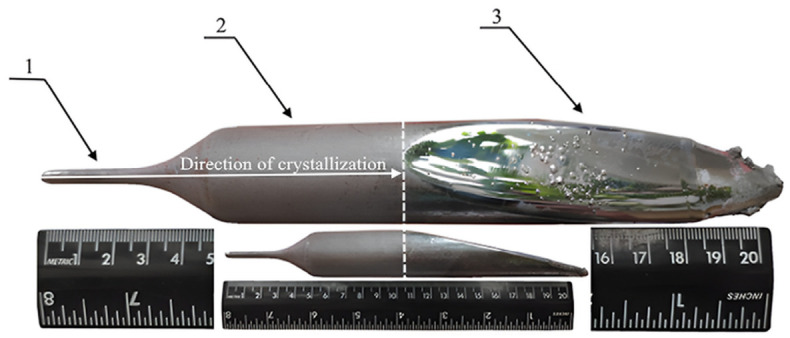
Photograph of the purified ingot after inclined purification, showing 1—seed; 2—ingot “body”; 3—the “tail” region and the separation line.

After purification, the polycrystalline ingot had a longitudinal conical shape with a clearly formed “tail” segment containing the rejected impurities, as shown in Figure 9. This tail section was removed and excluded from subsequent crystal-growth stages.

After three purification cycles, the retained mass was 182 g from an initial mass of 460 g, corresponding to an overall yield of 66.91%. Thus, the purification process involves a material-yield trade-off, but it provides a controlled way to reject impurity-rich tail regions before crystal growth.

The tail portions of the ingots, which are enriched with impurities, are not discarded but can be efficiently reprocessed.

The purification mechanism in directional crystallization is determined by the difference in impurity solubility in the liquid and solid phases, quantitatively described by the equilibrium distribution coefficient:

(12) $$k_{0}=\frac{C_{s}}{C_{l}\;}$$

where *C_s_* and *C_l_* are the impurity concentrations in the solid and liquid phases, respectively, at the solid–liquid interface.

*k*_0_ is the equilibrium distribution coefficient.

When *k*_0_ < 1, the impurity preferentially remains in the melt, which enables its displacement toward the “tail” of the ingot during sequential crystallization.

Under real crystallization conditions, the distribution coefficient becomes an effective one because of the presence of a diffusion boundary layer of thickness δ ahead of the solidification front, as well as the finite front velocity v. Axial mixing/rotation reduces δ, bringing the process closer to equilibrium. The quantitative relationship between the equilibrium and effective distribution coefficients is described by:

(13) $$keff=\frac{k_{0}}{k_{0}+\left(1-k_{0}\right)exp\left(-\frac{v\delta }{D}\right)}$$

where *k*eff—effective distribution coefficient;

v—crystallization front velocity;
D—impurity diffusion coefficient in the liquid phase;
δ—diffusion boundary-layer thickness [23].

In the context of inclined directional crystallization of ZGP, the key factors enhancing purification efficiency are:

ensuring a stable temperature gradient and controlled movement of the crystallization zone;

minimizing the diffusion layer (via axial rotation/mixing), which reduces impurity trapping by the solidification front;

forming a well-defined “tail” region that concentrates the rejected impurities, followed by its removal from the processing cycle.

The subsequent growth of ZGP single crystals was carried out using the vertical Bridgman method on an oriented seed (seed orientation: (100)), starting from the pre-synthesized polycrystalline material. The vertical growth process was implemented in crystal growth furnaces similar to those described in [18,19]. The furnace consisted of a series of annular heating modules and thermal-insulating spacers assembled into a single frame; each module was equipped with a resistive heater and temperature sensors.

**Figure 10:**
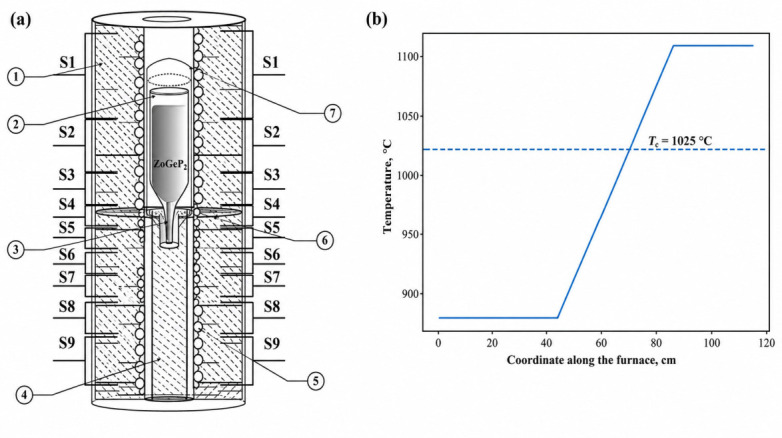
(**a**) Fragment of the working volume of the vertical Bridgman setup: 1—lining; 2—crucible; 3—seed; 4—stand; 5—resistive heaters; 6—diffuser; 7—ampoule; S1–S9—independently controlled temperature zones. (**b**) Scheme of the axial temperature distribution.

Within the working volume (a hollow cylinder approximately 6 cm in diameter), an axial temperature distribution with three zones was formed: a low-temperature zone (S8–S9), a high-temperature zone (S1–S3), and a gradient zone (S4–S7) (Figure 10). In the high-temperature zone, the polycrystal was melted and the seed was partially molten. During crystallization, the growth container with the melt was moved from the high-temperature zone into the low-temperature zone at a rate of approximately 5 mm/h.

A total of four ZGP single-crystal ingots were grown.

Ingot No. 1 was grown from polycrystalline material that had undergone the first purification cycle.

Ingot No. 2 was grown from material that had undergone the first and second purification cycles.

Ingot No. 3 was grown from material that had undergone the first, second, and third purification cycles.

Ingot No. 4 was grown from material that had not undergone inclined purification.

After the growth of the ZGP single crystals, control wafers were cut both along the crystal growth direction and perpendicular to it. The longitudinal sections had dimensions of 117 × 20 × 2 mm, while the transverse sections had a diameter of 30 mm and a thickness of 2 mm.

Polishing of the working surfaces of the samples was performed on a 4-PD-200 polishing and finishing machine (OAO Smorgon Optical Machine-Tool Plant, Smorgon, Belarus). The initial treatment of the sample surfaces consisted of polishing on a batiste polishing pad using synthetic diamond powder ACM 0.5/0 (average grain size 270 nm). In this step, approximately 50 μm of material was removed, which eliminated the cracked layer formed during cutting of the crystal into oriented plates and during preliminary grinding.

The samples were then additionally polished on a batiste pad using synthetic diamond powder ACM 0.25/0. After that, they were polished on a resin-based polishing pad made from polishing resin, again using synthetic diamond powder ACM 0.25/0.

## 4. Conclusions

A method of pre-growth purification of polycrystalline ZnGeP_2_ using inclined directional recrystallization—with formation and removal of a “tail” region containing rejected impurities and secondary phases—has been developed and tested.

It is shown that increasing the number of purification cycles improves the optical characteristics: the absorption coefficient at 2.097 µm decreases from 0.45 cm^−1^ to 0.3 cm^−1^ after three purification cycles.

The positive effect of repeated purification is confirmed by X-ray diffraction analysis: after the third recrystallization cycle, no intrinsic impurity contaminants (secondary phosphide phases) were detected in ZnGeP_2_ within the sensitivity of the PXRD method.

For quantitative evaluation of phase purity, a semi-quantitative metric based on the sum of integrated peak intensities (ΣI) is proposed: the impurity index Simp,I and the phase-purity index PurityI. According to this metric, the purity increases from 86.31% after the 1st purification to 95.995% after the 2nd, reaching 100% after the 3rd (within detection limits).

It is established that the increase in phase purity and reduction in absorption are accompanied by a decrease in LIDT with increasing number of pre-growth purification cycles; the likely cause is the accumulation of technological impurities during repeated high-temperature processing. This indicates the need for a compromise when selecting the number of purification cycles, depending on the target application (minimizing absorption vs. preserving LIDT). Another possible microscopic reason for the discrepancy between the reduction in absorption and the decrease in LIDT is the formation of point defects during repeated melting and recrystallization. Since ZnGeP_2_ contains volatile components Zn and P, repeated high-temperature treatment can locally disturb the stoichiometry and alter the defect equilibrium. This may lead to the formation of Zn and P vacancies, antisite defects, vacancy–antisite complexes, as well as donor–acceptor centers. Such defects may have little effect on the average infrared absorption coefficient, but under nanosecond laser irradiation, they can act as localized absorption centers. In this case, defect-associated absorption and the resulting local heating can initiate laser-induced damage even when the number of extended inclusions and crystalline secondary phases decreases.It should be noted that the LIDT values reported in this work were obtained for polished uncoated ZGP surfaces. Therefore, they characterize the combined influence of bulk crystal quality, polishing-induced surface/subsurface defects, and residual absorption near the surface. In practical OPO devices, ZGP crystals are often used with antireflection coatings. In such elements, the damage mechanism may additionally involve coating absorption, coating defects, thermal stress, and the coating-crystal interface. Consequently, LIDT values for uncoated and AR-coated ZGP crystals should be compared with caution, and coating optimization represents a separate technological problem.

## Figures and Tables

**Table 1:** Modes of pre-growth purification of ZnGeP_2_ (gradient, duration, number of cycles, charge mass, “tail” mass, yield and loss).

Loss (%)	Yield (%)	Tail Mass (g)	Charge Mass (g)	Rotation Speed (rpm)	Duration (h)	Temperature Gradient (°C/cm)	Purification Step
20.65	79.35	95	460	5	240	4	1
25.48	74.52	93	365	3	288	3	2
33.09	66.91	90	272	3	336	3	3
n/a	66.91	n/a	182	n/a	n/a	n/a	4

Note: n/a, not applicable.

## Data Availability

Data are contained within the article.

## Appendix A. PXRD Peak Lists Used for Semi-Quantitative Phase Assessment

This appendix reports the peak lists obtained from powder X-ray diffraction (PXRD) analysis for samples after different purification cycles. Values in parentheses denote the estimated standard uncertainty in the last digits as reported by the peak fitting software.

**Table A1:** PXRD peak list after the 1st purification cycle.

No.	2θ (deg)	d (Å)	Height	FWHM (deg)	Int. I	Size (Å)	Phase Name
1	20.67 (6)	4.294 (13)	1686 (119)	2.95 (10)	5293 (195)	28.6 (10)	Zinc Phosphide(1,1,−1)
2	21.124 (14)	4.202 (3)	4756 (199)	0.351 (16)	1779 (109)	240 (11)	Zinc Phosphide(2,0,0)
3	24.559 (12)	3.6219 (17)	11,225 (306)	0.32 (3)	4061 (358)	266 (25)	Zinc Phosphide(2,1,0)
4	29.71 (13)	3.005 (12)	7542 (251)	2.03 (15)	18,186 (1364)	42 (3)	Zinc Phosphide(1,3,1),Germanium Phosphide(3,0,0)
5	33.10 (6)	2.704 (5)	869,214 (2691)	0.40 (14)	373,784 (176,495)	214 (74)	Zinc Phosphide(2,2,0)
6	33.3633 (8)	2.68346 (6)	32,906,254 (16,560)	0.0954 (15)	3,343,223 (78,720)	908 (14)	Germanium Zinc Phosphide(2,0,0)
7	75.9354 (10)	1.252072 (13)	111,558 (964)	0.1200 (9)	17,450 (196)	877 (7)	Germanium Zinc Phosphide(3,3,2)
8	76.7219 (8)	1.241186 (11)	229,126 (1382)	0.1335 (7)	39,897 (205)	793 (4)	Germanium Zinc Phosphide(3,1,6)
9	58.935 (3)	1.56587 (7)	77,280 (802)	0.116 (2)	12,385 (140)	825 (17)	Germanium Zinc Phosphide(2,2,4)
10	34.07 (11)	2.630 (8)	84,183 (838)	1.2 (10)	104,167 (12820)	75 (65)	Zinc Phosphide(2,2, −1),Germanium Phosphide(2,1,1)
11	35.8 (8)	2.51 (5)	17,233 (379)	1.4 (3)	25,732 (5183)	62 (12)	Zinc Phosphide(0,2,2)
12	42.256 (13)	2.1370 (6)	6240 (228)	0.64 (3)	8431 (335)	139 (7)	Zinc Phosphide(1,3,1),Germanium Phosphide(3,0,0)

Note: Values in parentheses indicate the estimated standard uncertainty in the last digits.

**Table A2:** PXRD peak list after the 2nd purification cycle.

No.	2θ (deg)	d (Å)	Height	FWHM (deg)	Int. I	Size (Å)	Phase Name
1	18.266 (6)	4.8531 (16)	6162 (227)	0.195 (5)	1377 (38)	432 (11)	Germanium Zinc Phosphide(1,0,1)
2	27.387 (5)	3.2539 (6)	76,091 (796)	0.185 (15)	17,807 (935)	461 (37)	Germanium Phosphide(1,1,1)
3	28.490 (3)	3.1304 (3)	2,004,025 (4087)	0.132 (5)	413,627 (4051)	650 (26)	Germanium Zinc Phosphide(1,1,2)
4	32.796 (3)	2.7286 (2)	156,476 (1142)	0.149 (2)	29,955 (213)	581 (9)	Germanium Zinc Phosphide(2,0,0)
5	33.486 (5)	2.6739 (4)	49,368 (641)	0.167 (4)	10,035 (127)	520 (11)	Germanium Zinc Phosphide(0,0,4)
8	45.391 (7)	1.9964 (3)	62,164 (720)	0.129 (8)	9875 (401)	697 (45)	Germanium Phosphide(2,2,0)
9	47.019 (2)	1.93104 (10)	488,412 (2017)	0.126 (3)	93,398 (990)	717 (20)	Germanium Zinc Phosphide(2,2,0)
10	47.5541 (9)	1.91056 (3)	1,050,511 (2959)	0.1243 (10)	176,743 (606)	729 (6)	Germanium Zinc Phosphide(2,0,4)
11	53.790 (3)	1.70284 (8)	31,870 (515)	0.143 (3)	5938 (70)	653 (13)	Germanium Phosphide(3,1,1)
12	55.8946 (6)	1.643618 (16)	535,664 (2113)	0.1266 (6)	88,914 (874)	742 (4)	Germanium Zinc Phosphide(3,1,2)
13	56.8099 (10)	1.61929 (3)	195,789 (1277)	0.1427 (11)	36,645 (176)	661 (5)	Germanium Zinc Phosphide(1,1,6)
14	58.935 (3)	1.56587 (7)	77,280 (802)	0.116 (2)	12,385 (140)	825 (17)	Germanium Zinc Phosphide(2,2,4)
15	66.091 (3)	1.41260 (5)	16,766 (374)	0.126 (3)	2731 (60)	783 (18)	Germanium Phosphide(4,0,0)
20	68.6679 (9)	1.365736 (16)	133,936 (1056)	0.1180 (8)	20,441 (260)	852 (6)	Germanium Zinc Phosphide(4,0,0)
21	70.298 (2)	1.33800 (3)	30,506 (504)	0.157 (2)	6172 (59)	648 (9)	Germanium Zinc Phosphide(0,0,8)
22	72.923 (5)	1.29618 (8)	10,343 (294)	0.157 (4)	1724 (61)	659 (19)	Germanium Phosphide(3,3,1)
24	75.9354 (10)	1.252072 (13)	111,558 (964)	0.1200 (9)	17,450 (196)	877 (7)	Germanium Zinc Phosphide(3,3,2)
25	76.7219 (8)	1.241186 (11)	229,126 (1382)	0.1335 (7)	39,897 (205)	793 (4)	Germanium Zinc Phosphide(3,1,6)
26	78.172 (3)	1.22175 (4)	22,780 (436)	0.124 (3)	3896 (73)	861 (23)	Germanium Zinc Phosphide(4,2,0)
27	78.557 (3)	1.21673 (4)	24,153 (449)	0.129 (3)	4251 (73)	832 (21)	Germanium Zinc Phosphide(4,0,4)
28	79.717 (3)	1.20191 (3)	12,667 (325)	0.159 (3)	2413 (47)	678 (15)	Germanium Zinc Phosphide(2,0,8)
29	83.769 (2)	1.15377 (3)	22,164 (430)	0.135 (2)	3966 (44)	826 (15)	Germanium Phosphide(4,2,2)
32	87.7352 (11)	1.111548 (11)	201,512 (1296)	0.1309 (10)	34,446 (392)	880 (7)	Germanium Zinc Phosphide(4,2,4)
33	88.874 (3)	1.10023 (3)	79,799 (815)	0.164 (2)	15,701 (172)	708 (10)	Germanium Zinc Phosphide(2,2,8)

Note: Values in parentheses indicate the estimated standard uncertainty in the last digits.

**Table A3:** PXRD peak list after the 3rd purification cycle.

No.	2θ (deg)	d (Å)	Height	FWHM (deg)	Int. I	Size (Å)	Phase Name
1	18.301 (12)	4.844 (3)	700 (26)	0.193 (17)	168 (15)	436 (39)	Germanium Zinc Phosphide(1,0,1)
2	28.539 (2)	3.1251 (3)	156,985 (396)	0.166 (2)	34,558 (164)	516 (7)	Germanium Zinc Phosphide(1,1,2)
3	32.8199 (15)	2.72663 (12)	18,929 (138)	0.1391 (15)	3619 (14)	622 (7)	Germanium Zinc Phosphide(2,0,0)
4	33.527 (3)	2.6707 (2)	6084 (78)	0.153 (3)	1304 (11)	567 (13)	Germanium Zinc Phosphide(0,0,4)
5	47.0676 (10)	1.92917 (4)	50,752 (225)	0.1263 (11)	9077 (33)	716 (6)	Germanium Zinc Phosphide(2,2,0)

Note: Values in parentheses indicate the estimated standard uncertainty in the last digits.
